# Automatic segmentation of gadolinium-enhancing lesions in multiple sclerosis using deep learning from clinical MRI

**DOI:** 10.1371/journal.pone.0255939

**Published:** 2021-09-01

**Authors:** Sibaji Gaj, Daniel Ontaneda, Kunio Nakamura

**Affiliations:** 1 Department of Biomedical Engineering, Cleveland Clinic, Cleveland, Ohio, United States of America; 2 Mellen Center for Multiple Sclerosis, Cleveland Clinic, Cleveland, Ohio, United States of America; NIH, UNITED STATES

## Abstract

Gadolinium-enhancing lesions reflect active disease and are critical for in-patient monitoring in multiple sclerosis (MS). In this work, we have developed the first fully automated method to segment and count the gadolinium-enhancing lesions from routine clinical MRI of MS patients. The proposed method first segments the potential lesions using 2D-UNet from multi-channel scans (T1 post-contrast, T1 pre-contrast, FLAIR, T2, and proton-density) and classifies the lesions using a random forest classifier. The algorithm was trained and validated on 600 MRIs with manual segmentation. We compared the effect of loss functions (Dice, cross entropy, and bootstrapping cross entropy) and number of input contrasts. We compared the lesion counts with those by radiologists using 2,846 images. Dice, lesion-wise sensitivity, and false discovery rate with full 5 contrasts were 0.698, 0.844, and 0.307, which improved to 0.767, 0.969, and 0.00 in large lesions (>100 voxels). The model using bootstrapping loss function provided a statistically significant increase of 7.1% in sensitivity and of 2.3% in Dice compared with the model using cross entropy loss. T1 post/pre-contrast and FLAIR were the most important contrasts. For large lesions, the 2D-UNet model trained using T1 pre-contrast, FLAIR, T2, PD had a lesion-wise sensitivity of 0.688 and false discovery rate 0.083, even without T1 post-contrast. For counting lesions in 2846 routine MRI images, the model with 2D-UNet and random forest, which was trained with bootstrapping cross entropy, achieved accuracy of 87.7% using T1 pre-contrast, T1 post-contrast, and FLAIR when lesion counts were categorized as 0, 1, and 2 or more. The model performs well in routine non-standardized MRI datasets, allows large-scale analysis of clinical datasets, and may have clinical applications.

## Introduction

Multiple sclerosis (MS) is an inflammatory disease of the central nervous system and is characterized by the accumulation of lesions [1]. To identify active MS lesions, gadolinium-based contrast agents are administered to enhance contrast on T1-weighted magnetic resonance imaging (MRI) [2,3]. Gadolinium-based measures such as counts of gadolinium-enhancing lesions are used to monitor breakthrough disease and to evaluate the efficacy and effectiveness of anti-inflammatory agents in MS clinical trials and in clinical practice [4–6]. However, automated segmentation of gadolinium-enhancing lesions is challenging as their morphology differs greatly in size (for example, approximately 5 to > 100 voxels), shape (ring-like hollow and ovoid), intensity, and location. Earlier, Datta et al. [7] used morphological operations on T1 post-contrast images to find gadolinium-enhancing lesions and removed the false positive lesions by intensity-based thresholding, using the ratio of the difference between T1 post-contrast and T1 pre-contrast images to the T1 post-contrast images. Further, they used T2 images to remove false positives due to vasculature and structures such as the choroid plexus. Karimaghaloo et al. [8] have used a multi-label conditional random field to identify gadolinium-enhancing lesions from pre- and post-contrast T1-weighted images along with T2, PD, and FLAIR images. In that work, a relative enhancement in a T1 post-contrast image compared with a T1 pre-contrast image was used as an initial step. That approach required the same sequences for pre- and post-contrast images, which was not necessarily available in a clinical setting.

Recently, deep learning has been applied in medical image segmentations [9,10] and MS white matter lesion segmentations [11–14]. Narayana et al. [15] have used a deep learning-based network to predict lesion enhancement without administration of gadolinium-based contrast agents using T1, T2, and FLAIR images. Recently, Coronado et al. [16] have used 3D-UNet to segment gadolinium-enhancing lesions from pre- and post-contrast T1-weighted images along with T2, PD, and FLAIR images for a standardized clinical trial dataset.

Though several studies have been reported for segmenting gadolinium-enhancing lesions using a standardized dataset, there has not been a study using a non-standardized routine clinical dataset. Therefore, we propose a novel two-stage framework for segmentation and detection of gadolinium-enhancing MS lesions for a routine clinical MRI dataset. As UNet has performed well in lesion segmentations [16], we use 2D-UNet [17] to generate a voxel-wise probability map from multi-channel MRI scans in the first stage, then these probable lesions are filtered using a random forest-based classifier [18–20]. Several aspects of the proposed fully-automatic segmentation algorithm are assessed and reported. First, we evaluated the model performance with commonly-used Dice and cross entropy as well as bootstrapping cross entropy loss function. Then we tested different combinations of MRI input contrasts. The final trained model was applied to a separate clinical dataset to evaluate the accuracy of the gadolinium-enhancing lesion count.

## Methods

### Datasets

Two datasets were used, the first with manual lesion segmentation and the second with gadolinium-enhancing lesion counts. The first dataset (Dataset A) consisted of 600 routine scans from 496 patients with 5 contrasts (FLAIR, PD-weighted, T2-weighted, T1 pre-contrast, and T1 post-contrast). The mean (standard deviation [SD]) age was 42.7 (9.1) years, the mean disease duration was 10.2 (11.8) years, and the percentage of females was 74.1%. The scans were acquired as a part of routine practice in a single institution on various scanners at 1.0T, 1.5T, and 3T manufactured by Siemens, General Electrics, Picker, and Philips. The details of MRI sequence parameters are shown in Table 1. Gadolinium-enhancing lesions were segmented by a single rater with 17 years of experience in MS image analysis on preprocessed images. The manual segmentation was performed on co-registered images so that the hyperintensity on FLAIR and/or T2-weighted scans were verified. Furthermore, in order to identify and remove atypical vascular enhancement and persistent non-MS enhancement, the post-contrast scans were registered to those from the same subjects from different time points that were acquired at least six months apart. A total of 365 images from 262 patients contained at least one gadolinium-enhancing lesion. Images from patients with lesions were randomly split into 198 training subjects (301 images), 18 validation subject (18 images) and 46 testing subjects (46 images). The remaining 235 images without gadolinium-enhancing lesions were included in the test dataset. The ratio of training, validation, and testing subjects was approximately 40:5:55. These were routine MRIs without standardization where pre- and post-contrast T1-weighted scans may not use the same sequence parameters.

**Table 1:** Summary of MRI sequence parameters for manual segmentation dataset (Dataset A).

	MRI Sequences				
Parameters	T1-post-contrast	T1-pre-contrast	FLAIR	T2	PD
**Echo time (TE) (msec)**	2.46–20	2.46–20	75–140	72–166	8.18–36.0
**Repetition time (TR) (msec)**	28–773	28–1960	6000–11000	2000–7080	2000–7450
**Inversion time (TI msec)**	N/A	N/A	2000–2800	N/A	N/A
**Flip angle (degree)**	27–180	10–180	90–180	90–180	90–180
**Voxel size (mm)**	(0.41–1.0) x (0.41–1.0) x (3.0–6.5)	(0.41–1.19) x (0.41–1.19) x (1.0–6.5)	(0.41–1.0) x (0.41–1.0) x (3.0–6.5)	(0.27–1.0) x (0.27–1.0) x (3.0–6.5)	(0.27–1.0) x (0.27–1.0) x (3.0–6.5)
**Data acquisition format [format (number of scans)]**	2D Format (479), 3D Format (0), N/A (121)	2D Format (489), 3D Format (1), N/A (110)	2D Format (492), 3D Format (0), N/A (108)	2D Format (487), 3D Format (0), N/A (487)	2D Format (491), 3D Format (0), N/A (109)
**Vendors (number of scans)**	Siemens (516), GE Medical Systems(46), Philips Medical Systems(30), N/A (8)				
**Magnetic Field Strength (number of scans)**	1.0 T (37), 1.5 T (261), 3 T (192), N/A (10)				
**Total Subjects**	497				
**Total Scans**	600				

The parameters that were not available are marked N/A.

The second dataset (Dataset B) consisted of 2,846 routine images from 1,409 MS patients who are not in Dataset A. Dataset B had a mean (SD) age of 47.8 (11.7) years, disease duration of 12.1 (9.2) years, and a 73.3% female percentage. All subjects in this dataset had three MRI sequences: FLAIR, T1 pre-contrast, and T1 post-contrast images. The sequence parameters are shown on Table 2. Unlike Dataset A, which had manually segmented lesions, Dataset B contains the gadolinium-enhancing lesion count per image as provided in radiology reports assessed by board-certified radiologists. The reports categorized the counts as 0, 1, and 2 or above. The number of images with at least one gadolinium-enhancing lesion was 185; 2,661 scans did not have any gadolinium-enhancing lesions. The scans of both datasets were collected from Cleveland Clinic’s database under a single study approved by local IRB and de-identified before processing.

**Table 2:** Summary of MRI sequences parameters for lesion count dataset (Dataset B).

	MRI Sequences		
Parameters	T1-post-contrast	T1-pre-contrast	FLAIR
**Echo time (TE) (msec)**	2.46–17	1.71–17	77–393
**Repetition time (TR) (msec)**	11–1860	28–2300	4000–11710
**Inversion time (TI msec)**	N/A	N/A	1600–2800
**Flip angle (degree)**	10–90	8–90	90–180
**Voxel size (mm)**	(0.41–1.19) x (0.41–1.19) x (1–6.5)	(0.41–1.19) x (0.41–1.19) x (1–6.5)	(0.32–1) x (0.32–1) x (1–6.5)
**Data acquisition format [format (number of scans)]**	2D Format (2831), 3D Format (4), N/A (11)	2D Format (1187), 3D Format (1659), N/A (0)	2D Format (1193), 3D Format (1653), N/A (0)
**Vendors (number of scans)**	Siemens (2741), Philips Medical Systems (103), FONAR Corp. (2)		
**Magnetic Field Strength (number of scans)**	0.6 T (2), 1 T (30), 1.5 T (954), 3T (1860)		
**Total Subjects**	1409		
**Total Scans**	2846		

The parameters that were not available are marked N/A.

### Preprocessing

The analysis was performed in the linearly registered ICBM space. Standard preprocessing was applied and included N3 intensity non-uniformity correction [21], in-house intra-subject registration using normalized mutual information, standard space linear registration [22] with ICBM template [23], skull-extraction using brain extraction based on nonlocal segmentation technique [24], and intensity normalized by fitting to a standard ICBM template within the brain mask using MINC toolkit’s program volume_pol [25]. Template registration was further refined by ANTS nonlinear registration [26]. The template’s probability maps of gray matter, white matter, cerebrospinal fluid, lateral ventricles, and lesion from MINC toolkit [27] were nonlinearly transformed to the subject’s linear template space. Therefore, all images were linearly transformed in a standard space and resampled at 1x1x3mm resolution with 64 256x256 2D slices.

### Model specification

**Fig 1:**
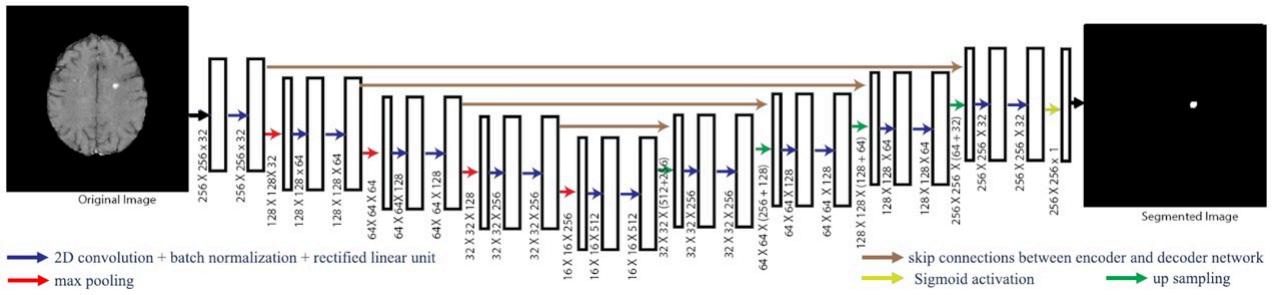
UNet architecture. The model has five depths of encoder and decoder. Blue arrows represent 2D convolution layers with batch normalization and rectified linear unit, red arrows represent max pooling layers, and green arrows represent up sampling layers. Brown arrows are skip connections between the encoder and decoder networks. MRI contrasts and probability maps are the input, and the segmented lesion map is the output.

We used 2D-UNet to segment the gadolinium-enhancing lesion from MRI images, as UNet has been shown to provide a state-of-the-art segmentation performance in biomedical image segmentation [10,17,28]. It contains encoder and decoder networks with skip connections to improve the information flow and preserve low-level spatial features [28]. The proposed UNet architecture is given in Fig 1. The algorithm uses 2D slices from co-registered MRI contrasts (FLAIR, PD-weighted, T2-weighted, T1 pre-contrast, and T1 post-contrast) and five probability maps (gray matter, white matter, cerebrospinal fluid, lateral ventricles, and lesion probability maps) as input, and produces a corresponding voxel-wise probability map for the gadolinium-enhancing lesions. The encoder and decoder consist of five depths, where each depth has two convolution layers followed by batch normalization and rectified linear unit (ReLU) activation. The number of filters in each depth of encoder is 32, 64, 128, 256, and 512. Similarly, the up-sampling decoder path has 512, 256, 128, 64, and 32 filters at each depth, respectively. The last layer of the decoder is followed by a sigmoid activation function. The total number of parameters for UNet is 7.8 million. The developed models and Python scripts used in this study are available in a public repository (https://github.com/sibajigaj/Gad_lesion_segmentation).

### Loss function

We have used three different loss functions and evaluated their performance: (a) Dice coefficient loss, (b) cross entropy, and (c) bootstrapped cross entropy. In the literature, Dice coefficient loss and binary cross entropy loss have provided state-of-the-art semantic segmentation performance in many domains [9,10], including MS white matter hyperintense lesion segmentation [11,14].

Let *x* be the manual segmentation mask, $\hat{x}$ the predicted segmentation mask with probability values for each voxel, *N* the total number of voxels and *j* the voxel index in the mask. Then the Dice coefficient loss (denoted by *L*_*seg_dice*_) can be written as

$$L_{seg\_dice}=[1-\frac{2\;\sum_{j=1}^{N}x_{j}\;{\hat{x}}_{j}\;}{\sum_{j=1}^{N}x_{j}+\;\sum_{j=1}^{N}\;{\hat{x}}_{j}\;}].$$ (1)

Similarly, the cross entropy loss for j^th^ voxel can be defined as

$$p_{j}=-x_{j}\;\text{log}\left({\hat{x}}_{j}\right)-(1-x_{j})\;\text{log}\left(1-{\hat{x}}_{j}\right)$$ (2)

The overall cross entropy loss (denoted by *L*_*seg_ce*_) can be written as

$$L_{seg\_ce}=\frac{1}{N}\sum_{j=1}^{N}p_{j}$$ (3)

Further, we have used bootstrapping cross entropy to emphasize the voxels that are hard to segment during the training [29,30]. These tend to be boundary voxels and those in small lesions. During the training, we select *K* voxels that have the least probability of being correctly labelled. The bootstrapped loss function over *K* voxel is written as

$$L_{seg\_bt}=\frac{1}{K}\sum_{j=1}^{N}1[p_{j}<t]p_{j}\;,$$ (4)

where **1** is a indicator function, i.e., **1**[*x*] = 1 if x is true and threshold probability *t* is chosen such that |*j* ∈ {1, …, *N*}: [*p*_*j*_ < *t*]| = *K*. During the training iterations, *t* is determined by sorting the loss and selecting *K* + 1 th loss as the threshold.

### Model training and validation

All training was performed using Dataset A. The proposed network was implemented using Keras version 2.2.2 [31] and the TensorFlow 1.10.0 [32] framework and was trained on the Owens Cluster with NVIDIA Tesla P100 GPU at the Ohio Supercomputer Center [33].

We trained the model separately using Dice coefficient loss, cross entropy loss alone and bootstrapping cross entropy loss with K = 256, 1,536 (= 256x6), and 3,072 (= 256x12). We used 10 channels from each 2D slice as an input and a manual lesion segmentation mask as the target output. The batch size was 16, and the ADAM optimizer (momentum value of 0.5 and a learning rate of 1e-4) was used. For each iteration, consecutive sets of 2D images in the slice direction formed a batch. As gadolinium-enhancing lesions are spatially sparse, most of the batches from training images did not have any lesion, from which the model only learned non-lesional features. Therefore, we altered the probability of training batches based on the presence of lesions by always using the batches with lesions and randomly excluding the batches without any lesions.

For validation and testing, 2D slices from datasets were individually input to the model and produced 2D lesion segmentation masks. The masks were stacked together to generate a 3D segmentation mask for each image set. The segmentation performance was evaluated as an average of 3D Dice coefficients from the predicted images.

### Post-processing with random forest

Similar to Elliott et al. [34] we added 3D post-processing using a random forest classifier to reduce the false detection rate, as 2D-UNet lacked 3D information. First, 2D-UNet was applied to predict initial segmentations, then 3D-connected components within the predicted segmentations were found using 6 connectivity. Next, we extracted 75 features for each of the connected 3D lesion candidates and trained the random forest. These features were nine lesion location features (x, y, z coordinates of the candidate center, starting x, y, z coordinates, depth in x, y, z axis), number of the candidates within the scan, five shape-based (size of the candidate in voxels, mean and standard deviation of eigenvalues, axial diffusivity and radial diffusivity of the candidate) and 60 intensity-based (mean, standard deviation, and sum of the intensity values within candidates’ objects and in their surrounding in each of the five MRI contrast and five probability maps). To train the random forest classifier, we used the training and validation data from Dataset A.

### Model performances evaluation

For evaluating the performance of our algorithm, we performed various tests: (a) comparisons of three loss functions (Dice, cross-entropy, and bootstrapped cross-entropy); (b) Dice similarity, lesion-wise sensitivity analysis using Dataset A; (c) accuracy of lesion counts using Dataset B, and; (d) effect of MRI contrasts.

In the context of segmenting gadolinium-enhancing lesions, delineating the correct boundary is difficult and has high inter- and intra-rater variability. Therefore, in addition to Dice coefficients, we used lesion-wise sensitivity and false discovery rate to evaluate the model performance from Dataset A. The detected lesions were identified as true positive (TP) if they had at least one voxel overlapping with the manual segmentation [8]. If the segmented lesion did not overlap with any part of manual segmentation, the lesion was classified as false positive (FP). If the manual segmentation did not overlap with any part of automatic segmentation, it was classified as false negative (FN). We used sensitivity (i.e. TP/(TP + FN)) and false detection ratio (FDR, i.e. FP/(FP + TP)) to evaluate lesion detection performance of the proposed model. Similar to Karimaghaloo et al [8], we evaluated the effect of gadolinium-enhancing lesion size on the model performance. We used a minimum lesion size of 5 voxels for all of our models. Finally, we calculated Dice for various size groups, as well as overall Dice from 3D image.

For Dataset B where categories of gadolinium-enhancing lesion counts (none, one and two or greater than two) are available, we automatically segmented lesions and counted them for each scan. The image is marked as accurately classified if the lesion count match between the proposed method and the radiologist’s provided a number. We presented a confusion matrix to show the model performance for each category. The diagonal elements represent the number of images for which the predicted group is equal to the true group, while off-diagonal elements are those that are misclassified by the proposed method. Along with the numbers of images in the confusion matrix, we also presented the normalized values by group size. We calculated overall accuracy, which is the percentage of correctly classified images within total assessed images. We have also evaluated performance variation for the magnetic field, scanner vendor and acquisition format (2D vs 3D) in Dataset B.

The effect of probability maps was assessed for the same measures (Dice, sensitivity, and false discovery rate) by comparing the model with and without the five probability maps. The effect of MRI contrasts was evaluated experimentally using the following combinations: (a) four contrasts (T1 post-contrast, T1 pre-contrast, T2-weighted and FLAIR scans); (b) three contrasts (T1 post-contrast, T2-weighed, and FLAIR); (c) three contrasts (T1 post-contrast, T1 pre-contrast, and FLAIR); (d) two contrasts (T1-post-contrast and FLAIR scans); (e) T1 post-contrast only; and finally (f) without T1 post-contrast (i.e., T1 pre-contrast, T2-weighted, PD-weighted, and FLAIR scans).

## Results

Table 3 shows the model performance with respect to sensitivity, FDR, and average Dice coefficients for the three functions from Dataset A. All UNet models used 10 input channels (five MRI contrasts and five probability maps). The highest sensitivity before random forest classifier was 0.916, which was achieved by the bootstrapping cross entropy (K = 256 X 12). For all of the loss function, FDR was improved after applying the RF. This improvement was statistically significant for all the loss function (*p* < 0.0001, using two-sided t-test). The change in sensitivity due to RF was also statistically significant for bootstrapping cross-entropy and Dice loss (*p* < 0.05, Supporting information).

**Table 3:** Model performance for test cases of Dataset A with different loss function.

Loss Function	Model	Lesion size (in Voxels)	5–10	11–20	21–50	51–100	>100	Total
		**Total lesion count**	21	41	49	36	32	179
**Dice coefficient loss**	UNet	TP Count	11	35	46	33	32	157
		Sensitivity	0.524	0.854	0.939	0.917	1.000	0.877
		FP Count	70	44	36	1	4	155
		FDR	0.864	0.557	0.439	0.029	0.111	0.497
		Dice*	0.165	0.465	0.521	0.691	0.772	0.655
	UNet + RF	TP Count	11	30	43	33	31	148
		Sensitivity	0.524	0.732	0.878	0.917	0.969	0.827
		FP Count	33	27	26	0	2	88
		FDR	0.75	0.474	0.377	0.000	0.061	0.373
		Dice	0.25	0.512	0.558	0.714	0.766	0.684
**Cross entropy**	UNet	TP Count	13	31	44	34	32	154
		Sensitivity	0.619	0.756	0.898	0.944	1	0.86
		FP Count	80	33	13	5	2	133
		FDR	0.86	0.516	0.228	0.128	0.059	0.463
		Dice	0.139	0.45	0.581	0.647	0.762	0.646
	UNet + RF	TP Count	9	28	41	32	31	141
		Sensitivity	0.429	0.683	0.837	0.889	0.969	0.788
		FP Count	25	17	8	1	1	52
		FDR	0.735	0.378	0.163	0.03	0.031	0.269
		Dice	0.225	0.523	0.587	0.686	0.753	0.682
**Bootstrapping cross entropy (K = 256)**	UNet	TP Count	13	35	43	34	32	157
		Sensitivity	0.619	0.854	0.878	0.944	1.000	0.877
		FP Count	48	20	12	1	3	84
		FDR	0.787	0.364	0.218	0.029	0.086	0.349
		Dice	0.195	0.475	0.582	0.736	0.702	0.646
	UNet + RF	TP Count	11	26	40	32	31	140
		Sensitivity	0.524	0.634	0.816	0.889	0.969	0.782
		FP Count	18	10	9	1	1	39
		FDR*	0.621	0.278	0.184	0.03	0.031	**0.218**
		Dice	0.294	0.503	0.589	0.727	0.727	0.678
**Bootstrapping cross entropy (K = 256 X 12)**	UNet	TP Count	14	38	46	34	32	164
		Sensitivity	0.667	0.927	0.939	0.944	1	0.916
		FP Count	106	36	20	4	1	167
		FDR	0.883	0.486	0.303	0.105	0.03	0.505
		Dice	0.147	0.52	0.601	0.692	0.775	0.661
	UNet + RF	TP Count	12	33	42	33	31	151
		Sensitivity*	0.571	0.805	0.857	0.917	0.969	**0.844**
		FP Count	31	17	15	4	0	67
		FDR	0.721	0.34	0.263	0.108	0	0.307
		Dice*	0.27	0.603	0.615	0.687	0.767	**0.698**

The input of the model is T1 post-contrast, T1 pre-contrast, FLAIR, T2, PD MRI sequences with five probability maps. The best performance is marked in bold. The statistically significant improvements compared to cross-entropy loss have been mark as *.

The model trained with bootstrapping cross entropy (K = 256 X 12) had the highest sensitivity of 0.844 and highest Dice of 0.698 after applying random forest classification. These improvements were statistically significant when compared to the model trained with cross entropy loss (p < 0.05). Dice and FDR significantly improved using the Dice loss and bootstrapped cross-entropy loss compared to the cross-entropy loss function (S4 Table). The model performance was the highest for largest lesions size group (> 100 voxels) within the respective loss functions. The maximum sensitivity of 0.969 and Dice of 0.767 were achieved by bootstrapping cross entropy (K = 256 X 12) with the largest lesion sizes. The random forest classifier affected each loss function differently. The smallest sensitivity decrease was 0.05 for Dice loss, and the largest was 0.11 for bootstrapping cross entropy loss. On the other hand, the largest decrease in FDR was 0.260 with cross entropy, and the smallest with bootstrapping cross entropy loss (K = 256 x 6). The importance features in the random forest classifier differed, but the consistently important features were the slice location, mean intensity on T1 post-contrast image within lesion, intensity variance on T1 post-contrast surrounding lesion, and sum of lesion probability within the gadolinium-enhancing lesion. The detailed experiment results with bootstrapping cross entropy are shown in S1 Table.

**Fig 2:**
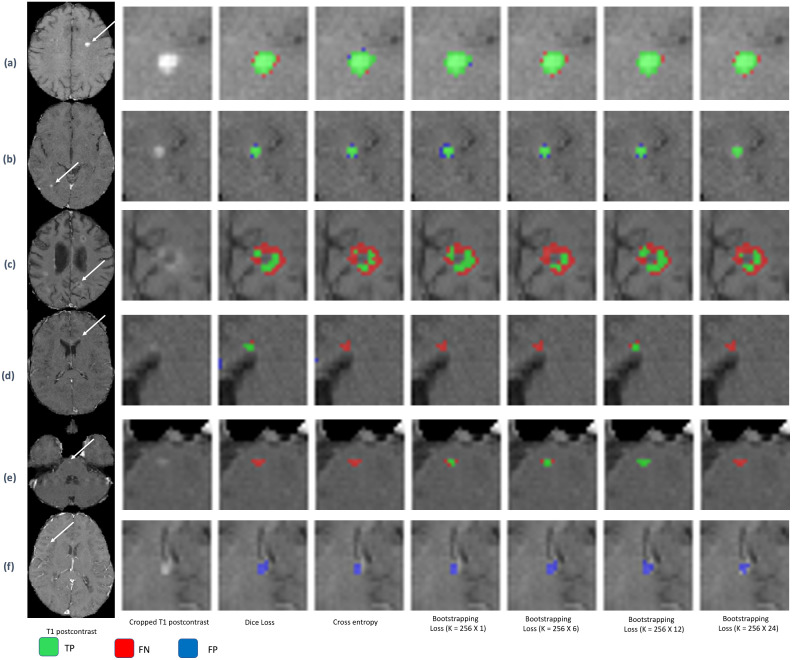
Predicted images for different loss functions. Each row represents image slices from different MRI sequences. The first column represents T1 post-contrast images and the second column represents the enlarged T1 post-contrast images around the lesion of interest. The other columns are performances of UNet and random forest model for different loss functions. Red regions are false negative segmentation, green areas are true positive, and blue regions are false positive regions. The white arrows are used to identify the lesion of interest. The first two rows (a and b) show example of lesions which are identified by all loss functions. The third row (c) shows change of lesion segmentation volume by loss functions. Fourth and fifth rows (d and e) show the improvement of segmentation due to bootstrapping loss function. The last row (f) shows example of false detection.

Fig 2 shows examples of lesion segmentation results for different loss functions. The first column in Fig 2 represents T1 post-contrast images and the second column represents the enlarged T1 post-contrast images around the lesion of interest. The other columns are performances of UNet and random forest model for different loss functions. Each row represents image slices from different MRI scans. Red regions are false negative segmentation, green areas area true positive, and blue regions are false positive regions. The white arrows are used to identify the lesion of interest. The first two rows (Fig 2a and 2b) show example of lesions that are identified by all loss functions. Fig 2d and 2e show examples of improvement in lesion segmentation in the bootstrapping loss. Fig 2f shows a false detection of lesion.

**Fig 3:**
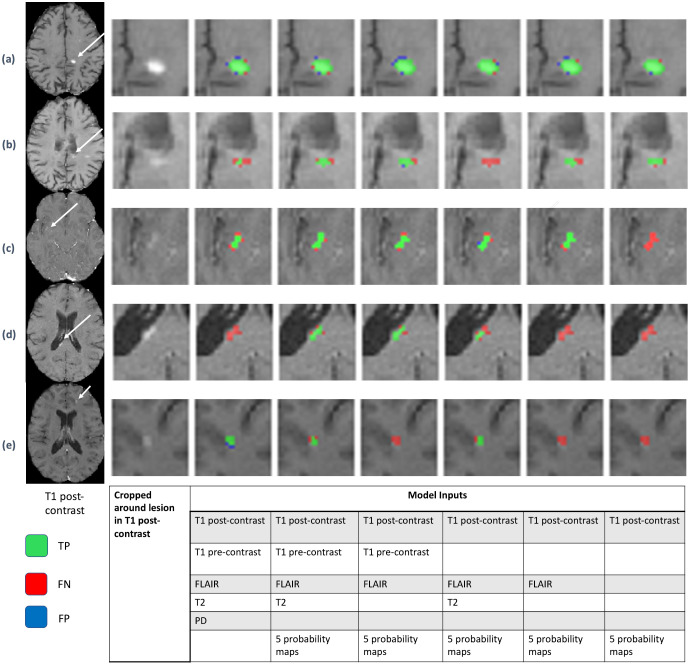
Predicted images for different input sequences. Each row represents image slices from different MRI sequences. The first column represents T1 post-contrast images and the second column represents the enlarged T1 post-contrast images around the lesion of interest. The other columns are performances of UNet and random forest model for different input sequences, which are tabulated below. The red regions are false negative segmentation, green is an overlap, and blue regions are false positives. The white arrow shows the lesion of interest. The first row (a) shows an example of lesion detection using any input while the second row (b) shows change in lesion segmentation volume due to different input. The third row (c) shows a lesion that cannot be detected using only T1 post-contrast. The fourth row (d) shows an example of lesion detection where T1 pre-contrast, T1 post-contrast and FLAIR are most influential to detect. The fifth row (e) shows a lesion that cannot be detected without T2.

Table 4 shows the effect of input contrasts on Dice accuracy, sensitivity, and FDR. These models were trained with the loss function of bootstrapping cross entropy, which has out-performed other cost functions. The sensitivity and FDR were similar as long as T1 post-contrast, T1 pre-contrast, and FLAIR were used as inputs. The sensitivity decreased to 0.777 from 0.844 when the probability maps were removed. Fig 3 shows examples of lesion segmentation results for different input sequences. Each row in Fig 3 represents image slices from different MRI scans. The first column represents T1 post-contrast images. The other columns are performances of UNet and random forest model for different input sequences, which are tabulated below within Fig 3. Fig 3a shows an example of lesion detection using any input, while Fig 3b shows a change in lesion segmentation volume due to a different input. Fig 3c shows a lesion which cannot be detected using only T1 post-contrast. Fig 3d shows an example of lesion detection where T1 pre-contrast, T1 post-contrast and FLAIR are most influential to detect. Fig 3e shows a lesion which cannot be detected without T2.

**Table 4:** Effect of different input combinations of MRI sequences from test cases of Dataset A.

Input Sequences (Number of contrasts)	Model	GAD size (in Voxels)	5–10	11–20	21–50	51–100	>100	Total
		**TotalGAD**	21	41	49	36	32	179
**T1 pre-contrast, T1 post-contrast, FLAIR, T2, PD (5)**	UNet	TP Count	13	35	44	34	32	158
		Sensitivity	0.524	0.854	0.939	0.917	1	0.883
		FP Count	39	23	7	0	1	70
		FDR	0.75	0.397	0.137	0	0.03	0.307
		Dice	0.201	0.555	0.585	0.780	0.752	0.689
	UNet + RF	TP Count	9	28	39	31	32	139
		Sensitivity	0.524	0.732	0.878	0.917	0.969	0.777
		FP Count	24	14	5	0	1	44
		FDR	0.727	0.333	0.114	0	0.03	0.24
		Dice	0.237	0.571	0.576	0.746	0.751	0.692
**T1 pre-contrast, T1 post-contrast, FLAIR, T2 and GM, WM, CSF, Ventricles, lesion probability (9)**	UNet	TP Count	12	35	45	34	32	158
		Sensitivity	0.571	0.854	0.918	0.944	1	0.883
		FP Count	32	13	7	1	1	54
		FDR	0.727	0.271	0.135	0.029	0.03	0.255
		Dice	0.238	0.562	0.580	0.765	0.773	0.698
	UNet + RF	TP Count	11	35	39	33	32	150
		Sensitivity	0.524	0.854	0.796	0.917	1	0.838
		FP Count	22	13	6	1	1	43
		FDR	0.667	0.271	0.133	0.029	0.03	0.223
		Dice	0.260	0.584	0.562	0.758	0.772	0.700
**T1 pre-contrast, T1 post-contrast, FLAIR and GM, WM, CSF, Ventricles, lesion probability (8)**	UNet	TP Count	13	35	44	34	32	158
		Sensitivity	0.619	0.854	0.898	0.944	1	0.883
		FP Count	28	23	6	2	1	60
		FDR	0.683	0.397	0.12	0.056	0.03	0.275
		Dice	0.274	0.548	0.575	0.708	0.768	0.689
	UNet + RF	TP Count	10	30	42	33	32	147
		Sensitivity	0.476	0.732	0.857	0.917	1	0.821
		FP Count	19	14	3	2	1	39
		FDR	0.655	0.318	0.067	0.057	0.03	0.21
		Dice	0.292	0.580	0.579	0.707	0.766	0.696
**T1 post-contrast, FLAIR, T2 and GM, WM, CSF, Ventricles, lesion probability (8)**	UNet	TP Count	11	32	45	34	32	154
		Sensitivity	0.524	0.78	0.918	0.944	1	0.86
		FP Count	48	29	7	1	1	86
		FDR	0.814	0.475	0.135	0.029	0.03	0.358
		Dice	0.163	0.457	0.638	0.706	0.766	0.681
	UNet + RF	TP Count	6	28	42	33	32	141
		Sensitivity	0.286	0.683	0.857	0.917	1	0.788
		FP Count	34	25	7	1	1	68
		FDR	0.85	0.472	0.143	0.029	0.03	0.325
		Dice	0.127	0.459	0.630	0.704	0.765	0.685
**T1 post-contrast, FLAIR and GM, WM, CSF, Ventricles, lesion probability (7)**	UNet	TP Count	16	36	45	33	32	162
		Sensitivity	0.762	0.878	0.918	0.917	1	0.905
		FP Count	88	38	19	2	1	148
		FDR	0.846	0.514	0.297	0.057	0.03	0.477
		Dice	0.168	0.483	0.586	0.701	0.760	0.652
	UNet + RF	TP Count	11	34	40	32	31	148
		Sensitivity	0.524	0.829	0.816	0.889	0.969	0.827
		FP Count	20	19	9	2	1	51
		FDR	0.645	0.358	0.184	0.059	0.031	0.256
		Dice	0.309	0.584	0.605	0.695	0.743	0.685
**T1 post-contrast and GM, WM, CSF, Ventricles, lesion probability (6)**	UNet	TP Count	11	31	41	34	32	149
		Sensitivity	0.524	0.756	0.837	0.944	1	0.832
		FP Count	71	31	11	4	0	117
		FDR	0.866	0.5	0.212	0.105	0	0.44
		Dice	0.128	0.416	0.505	0.606	0.781	0.627
	UNet + RF	TP Count	6	25	37	31	32	131
		Sensitivity	0.286	0.61	0.755	0.861	1	0.732
		FP Count	27	12	3	3	0	45
		FDR	0.818	0.324	0.075	0.088	0	0.25
		Dice	0.146	0.492	0.518	0.604	0.780	0.656
**T1 pre-contrast, FLAIR, T2, PD and GM, WM, CSF, Ventricles, lesion probability (9)**	UNet	TP Count	2	10	13	14	22	61
		Sensitivity	0.095	0.244	0.265	0.389	0.688	0.341
		FP Count	146	69	40	9	2	266
		FDR	0.986	0.873	0.755	0.391	0.083	0.813
		Dice	0.003	0.036	0.076	0.194	0.270	0.166
	UNet + RF	TP Count	0	4	9	12	18	43
		Sensitivity	0.000	0.098	0.184	0.333	0.563	0.240
		FP Count	5	4	2	0	0	11
		FDR	1.000	0.500	0.182	0.000	0.000	0.204
		Dice	0.000	0.055	0.105	0.228	0.271	0.217

Bootstrapped cross entropy was used to train these models.

For Dataset B with lesion count data, the model with bootstrapping cross entropy for three contrasts (T1 post-contrast, T1 pre-contrast, and FLAIR), five probability maps, and the random forest classifier, was used. The automatically segmented gadolinium-enhancing lesions were counted for each image and categorized into count groups. The lesion categorization is shown in Table 5, where TP and accuracy are presented as a confusion matrix. The overall accuracy for classification was 87.7%.

**Table 5:** Confusion matrix lesion count results on Dataset B.

		**True lesion count**		
		**0 lesion count**	**1 lesion count**	**≥2 lesion count**
**Predicted lesion count**	**0 lesion count**	2381 (89.5%)	38 (34.9%)	8 (10.5%)
	**1 lesion count**	234 (8.8%)	64 (58.7%)	16 (21.1%)
	**≥2 lesion count**	46 (1.7%)	7 (6.4%)	52 (68.4%)
**overall accuracy**		87.7%		

The diagonal elements represent the number of images for which the predicted lesion count is equal to the true lesion count.

S5 and S6 Tables show the model performance for different magnetic fields (1, 1.5, and 3T) and scanner vendors (Siemens and Philips), respectively. The overall accuracy for the 3T scans was the highest. The percentage of false negatives for the groups with enhancing lesions (1 lesion count group and ≥2 lesion count group) were lower in 1.5T scans compared to 3T scans: 38.1% vs 43.9% for 1 lesion count group and 19.4% vs. 38.4% for ≥2 lesion count group. For the vendor comparison, Siemens scanners had higher overall accuracy compared to Philips, but there was a limited number of scans with enhancing lesions from Philips scanners.

The performance comparison on the image acquisition format is given in S7 and S8 Tables. For T1 pre-contrast sequences, the overall accuracy for 3D acquisition was higher than 2D, but the percentage of false negatives for the groups with enhancing lesions was lower in 2D acquisitions: 36.7% vs 45% for 1 lesion count group and 19% vs 41.2% for ≥2 lesion count group. The finding was consistent for the FLAIR sequence (S8 Table). For Dataset B, most the scans for T1 post-contrast were acquired in the 2D format; only four scans were acquired in 3D, and none had lesions. Finally, we compared the scans where the sequence differed between pre- and post-contrast T1 versus those with the same sequence (S9 Table). The overall accuracy for scans with different sequences was higher, but the percentage of false negatives was smaller for the groups with enhancing lesions in the scans with same sequence: 35.9% vs 44.3% for 1 lesion count group and 21.1% vs 36.8% for ≥2 lesion count group.

## Discussion

We have presented a novel automated gadolinium-enhancing lesion segmentation pipeline from multi-sequence MRI scans acquired for routine practice in MS patients. Fully automated MS lesion segmentation is a very difficult task, as lesion size, shape, and location are highly variable. Additionally, these routine MRI scans have non-standardized sequence protocols with varying image contrast, signal intensity, and image artifacts. Finally, large inter- and intra-rater variability in manual segmentation poses a challenge in developing algorithms with a high accuracy.

In the proposed method, we first segmented the potential gadolinium-enhancing lesion from multi-sequence MRI scans using UNet. Then these lesions are filtered using a random forest classifier to reduce the FP. The UNet models using Dice loss, cross entropy loss, and bootstrapping cross entropy loss were compared. Overall, Dice was similar among different loss functions, and the sensitivity had a larger variance. The UNet alone with bootstrapping achieved highest sensitivity. As bootstrapping loss focuses more on the difficult voxels during training, it helped to detect small lesions compared to other loss functions and increased the sensitivity. The FDR is also competitively lower for bootstrapping loss. The same model achieved FDR of 0.307. Approximately 50% of these FP contrast-enhancing lesions belong to the smallest lesion size group (5–10 voxels).

There is a limited literature on methods to segment and measure gadolinium-enhancing lesion volume and counts. As there is no publicly-available dataset, every study thus far used different datasets to test their algorithms. Therefore, we cannot perform direct comparison to other methods. In terms of the datasets, the key difference from others is that we used routinely acquired MRIs from clinical practice, whereas several others used the images from clinical trials [8,15,35]. In our routinely collected dataset, where patients are well managed, the percentage of scans with contrast-enhancing lesions was low (around 6.5%) compared to the baseline values from typical RRMS clinical trials (>20%) [35–44], and thus such clinical trial datasets may provide large volumes of gold standards for training datasets, but may be less useful when attempting to implement algorithms in clinical settings. Since false positives are common, datasets with more scans with active lesions may show higher accuracy and sensitivity. To account for such problems, we have enriched the training datasets with patients with gadolinium-enhancing lesions and added patients without lesions in the testing datasets to reflect the general MS population. Another major difference in our non-trial dataset is the extent of standardization and quality assurance. For example, many of our images used different sequences for pre and post-contrast T1-weighted scans.

To compare our method with an existing method, we trained and tested a 3D-UNet, similar to the method by Coronado et al. [16]) on Dataset A. The sensitivity was comparable between the 3D-UNet (0.816) and our pipeline (0.844) in our dataset (S2 Table). Despite the reduced dimension, our 2D UNet model has an advantage in that the ratio of model parameters to input data size can be high; our 2D model has a ratio of 11.9, while the equivalent 3D model has only 1.8. This higher ratio of model parameters may have allowed the 2D-UNet to learn the structure and location of the lesions from 2D slices more efficiently. Additionally, the post-processing using the random forest includes the features in all three dimensions and likely improved our model performance. Another potential advantage of the proposed method is that it is robust against the variation in slice thickness and can be applied to T1 post-contrast scans with the 3D format. As the proposed method uses individual slices as input, it can be applicable to 3D-acquired T1 post-contrast images by resampling the images at a higher 1mm slicing or performing slice averaging (or maximum intensity projection) to create similar images as in the current training dataset.

We have also tested our model with different numbers of input sequences. The probability maps had a significant influence on the results. Clear lesions were segmented from reduced numbers of input contrasts, or even from a single post-contrast image as shown in Fig 3a. Overall, the algorithm’s performance was similar (sensitivity 0.821 and FDR 0.21) as long as T1 post-contrast, T1 pre-contrast, and FLAIR are present, compared to the full inputs (sensitivity 0.844 and FDR 0.263).

One limitation is that the retrospective nature of the study, where the count data does not contain location information; therefore, we cannot confirm that the algorithm has accurately segmented corresponding lesions. Also, as it was clinically acquired dataset, most of the scans did not have gadolinium-enhancing lesions. Thus, the overall accuracy was affected by class imbalance. The model can benefit from improvement, especially for the scans with a single gadolinium-enhancing lesion. As the lesion size can be very small and the dataset consists of heterogeneous scans, these scans are most challenging to detect by automatic methods. Potential improvements can be achieved by inclusion of more manual segmentation from multiple radiologists and by using custom model architectures. Another limitation is the reduced use of gadolinium-based contrast agents in light of recent findings on gadolinium accumulation in human brains [45,46]. We have tested without T1 post-contrast as a proof-of-concept. Average sensitivity, FDR and Dice were all low, thus suggesting machine learning of disease activity without administration of gadolinium contrast agent was not possible in this study. However, for large lesions (>100 voxels), the UNet was able to identify without T1 post-contrast image at reasonable sensitivity, suggesting detection for larger lesions may be feasible with larger training datasets.

## Conclusion

We demonstrate a two-stage deep learning-based framework to segment the active lesions from non-standardized routine MRI scans. To our knowledge, the proposed method is the first to develop a fully automatic lesion counting pipeline for such a non-standardized dataset. The UNet segmentation performance increases using bootstrapping cross entropy loss along with tissue probability maps. The proposed model achieved 0.844 sensitivity and 0.304 FDR where the proportion of images with enhancing lesions was rather small (15%). Our proposed network is flexible and has a potential to directly incorporate 3D information and dense network layers and include other tissue types.

## Supporting information

S1 Table Model performance for test cases of Dataset A with different loss function. The input of the model is T1 post-contrast, T1 pre-contrast, FLAIR, T2, PD MRI sequences with five probability maps. The best performance is marked in bold.
(DOCX)

S2 Table Performance of 3D UNet-based segmentation model on same test dataset of Dataset A. (DOCX)

S3 Table p-values for gadolinium-enhancing lesion detection between 2D-UNet and 2D-Unet + RF using different loss functions. (DOCX)

S4 Table p-values for gadolinium-enhancing lesion detection for 2D-Unet + RF between cross entropy loss and other loss functions. (DOCX)

S5 Table Confusion matrix lesion count results on Dataset B for different magnetic fields. The diagonal elements represent the number of images for which the predicted lesion count is equal to the true lesion count.
(DOCX)

S6 Table Confusion matrix lesion count results on Dataset B for different vendors. The diagonal elements represent the number of images for which the predicted lesion count is equal to the true lesion count.
(DOCX)

S7 Table Confusion matrix lesion count results on Dataset B for different acquisition formats of T1 pre-contrast sequences. The diagonal elements represent the number of images for which the predicted lesion count is equal to the true lesion count.
(DOCX)

S8 Table Confusion matrix lesion count results on Dataset B for different acquisition formats of FLAIR sequences. The diagonal elements represent the number of images for which the predicted lesion count is equal to the true lesion count.
(DOCX)

S9 Table Confusion matrix lesion count results on Dataset B for different acquisition formats of FLAIR sequences. The diagonal elements represent the number of images for which the predicted lesion count is equal to the true lesion count.
(DOCX)
