# Supplementary material for: Automatic segmentation of gadolinium-enhancing lesions in multiple sclerosis using deep learning from clinical MRI
Source: PLoS One. 2021 Sep 1;16(9):e0255939. doi: 10.1371/journal.pone.0255939 (PMC8409666; doi:10.1371/journal.pone.0255939)
Supplement: S1 Table — The input of the model is T1 post-contrast, T1 pre-contrast, FLAIR, T2, PD MRI sequences with five probability maps. The best performance is marked in bold. (DOCX) [file pone.0255939.s001.docx]

**Supplementary Table 1: Model performance for test cases of Dataset A with different loss function. The input of the model is T1 post-contrast, T1 pre-contrast, FLAIR, T2, PD MRI sequences with five probability maps. The best performance is marked in bold.**

| Loss Function | Model | Lesion size (in voxels) | 5-10 | 11-20 | 21-50 | 51-100 | >100 | Total |
| --- | --- | --- | --- | --- | --- | --- | --- | --- |
|  |  | **Total lesion count** | 21 | 41 | 49 | 36 | 32 | 179 |
| Dice  coefficient  loss | UNet | TP Count | 11 | 35 | 46 | 33 | 32 | 157 |
|  |  | Sensitivity | 0.524 | 0.854 | 0.939 | 0.917 | 1.000 | 0.877 |
|  |  | FP Count | 70 | 44 | 36 | 1 | 4 | 155 |
|  |  | FDR | 0.864 | 0.557 | 0.439 | 0.029 | 0.111 | 0.497 |
|  |  | Dice | 0.165 | 0.465 | 0.521 | 0.691 | 0.772 | 0.655 |
|  | UNet + RF | TP Count | 11 | 30 | 43 | 33 | 31 | 148 |
|  |  | Sensitivity | 0.524 | 0.732 | 0.878 | 0.917 | 0.969 | 0.827 |
|  |  | FP Count | 33 | 27 | 26 | 0 | 2 | 88 |
|  |  | FDR | 0.75 | 0.474 | 0.377 | 0.000 | 0.061 | 0.373 |
|  |  | Dice | 0.25 | 0.512 | 0.558 | 0.714 | 0.766 | 0.684 |
| Cross entropy | UNet | TP Count | 13 | 31 | 44 | 34 | 32 | 154 |
|  |  | Sensitivity | 0.619 | 0.756 | 0.898 | 0.944 | 1 | 0.86 |
|  |  | FP Count | 80 | 33 | 13 | 5 | 2 | 133 |
|  |  | FDR | 0.86 | 0.516 | 0.228 | 0.128 | 0.059 | 0.463 |
|  |  | Dice | 0.139 | 0.45 | 0.581 | 0.647 | 0.762 | 0.646 |
|  | UNet + RF | TP Count | 9 | 28 | 41 | 32 | 31 | 141 |
|  |  | Sensitivity | 0.429 | 0.683 | 0.837 | 0.889 | 0.969 | 0.788 |
|  |  | FP Count | 25 | 17 | 8 | 1 | 1 | 52 |
|  |  | FDR | 0.735 | 0.378 | 0.163 | 0.03 | 0.031 | 0.269 |
|  |  | Dice | 0.225 | 0.523 | 0.587 | 0.686 | 0.753 | 0.682 |
| Bootstrapping  cross entropy  (K= 256) | UNet | TP Count | 13 | 35 | 43 | 34 | 32 | 157 |
|  |  | Sensitivity | 0.619 | 0.854 | 0.878 | 0.944 | 1.000 | 0.877 |
|  |  | FP Count | 48 | 20 | 12 | 1 | 3 | 84 |
|  |  | FDR | 0.787 | 0.364 | 0.218 | 0.029 | 0.086 | 0.349 |
|  |  | Dice | 0.195 | 0.475 | 0.582 | 0.736 | 0.702 | 0.646 |
|  | UNet + RF | TP Count | 11 | 26 | 40 | 32 | 31 | 140 |
|  |  | Sensitivity | 0.524 | 0.634 | 0.816 | 0.889 | 0.969 | 0.782 |
|  |  | FP Count | 18 | 10 | 9 | 1 | 1 | 39 |
|  |  | FDR | 0.621 | 0.278 | 0.184 | 0.03 | 0.031 | **0.218** |
|  |  | Dice | 0.294 | 0.503 | 0.589 | 0.727 | 0.727 | 0.678 |
| Bootstrapping  cross entropy  (K= 256 X 6) | UNet | TP Count | 12 | 31 | 43 | 34 | 32 | 152 |
|  |  | Sensitivity | 0.571 | 0.756 | 0.878 | 0.944 | 1 | 0.849 |
|  |  | FP Count | 50 | 19 | 18 | 3 | 4 | 94 |
|  |  | FDR | 0.806 | 0.38 | 0.295 | 0.081 | 0.111 | 0.382 |
|  |  | Dice | 0.184 | 0.514 | 0.542 | 0.744 | 0.723 | 0.657 |
|  | UNet + RF | TP Count | 10 | 22 | 38 | 31 | 31 | 132 |
|  |  | Sensitivity | 0.476 | 0.537 | 0.776 | 0.861 | 0.969 | 0.737 |
|  |  | FP Count | 23 | 12 | 10 | 1 | 2 | 48 |
|  |  | FDR | 0.697 | 0.353 | 0.208 | 0.031 | 0.061 | 0.267 |
|  |  | Dice | 0.259 | 0.519 | 0.552 | 0.738 | 0.737 | 0.681 |
| Bootstrapping  cross entropy  (K= 256 X 12) | UNet | TP Count | 14 | 38 | 46 | 34 | 32 | 164 |
|  |  | Sensitivity | 0.667 | 0.927 | 0.939 | 0.944 | 1 | 0.916 |
|  |  | FP Count | 106 | 36 | 20 | 4 | 1 | 167 |
|  |  | FDR | 0.883 | 0.486 | 0.303 | 0.105 | 0.03 | 0.505 |
|  |  | Dice | 0.147 | 0.52 | 0.601 | 0.692 | 0.775 | 0.661 |
|  | UNet + RF | TP Count | 12 | 33 | 42 | 33 | 31 | 151 |
|  |  | Sensitivity | 0.571 | 0.805 | 0.857 | 0.917 | 0.969 | **0.844** |
|  |  | FP Count | 31 | 17 | 15 | 4 | 0 | 67 |
|  |  | FDR | 0.721 | 0.34 | 0.263 | 0.108 | 0 | 0.307 |
|  |  | Dice | 0.27 | 0.603 | 0.615 | 0.687 | 0.767 | **0.698** |
| Bootstrapping  cross entropy  (K= 256 X 24) | UNet | TP Count | 11 | 34 | 45 | 35 | 32 | 157 |
|  |  | Sensitivity | 0.524 | 0.829 | 0.918 | 0.972 | 1.000 | 0.877 |
|  |  | FP Count | 76 | 22 | 12 | 3 | 1 | 114 |
|  |  | FDR | 0.874 | 0.393 | 0.211 | 0.079 | 0.030 | 0.421 |
|  |  | Dice | 0.123 | 0.455 | 0.582 | 0.708 | 0.778 | 0.668 |
|  | UNet + RF | TP Count | 7 | 27 | 41 | 33 | 32 | 140 |
|  |  | Sensitivity | 0.333 | 0.659 | 0.837 | 0.917 | 1.000 | 0.782 |
|  |  | FP Count | 30 | 10 | 11 | 3 | 1 | 55 |
|  |  | FDR | 0.811 | 0.270 | 0.212 | 0.083 | 0.030 | 0.282 |
|  |  | Dice | 0.177 | 0.470 | 0.575 | 0.706 | 0.777 | 0.690 |
