# Supplementary material for: Automatic segmentation of gadolinium-enhancing lesions in multiple sclerosis using deep learning from clinical MRI
Source: PLoS One. 2021 Sep 1;16(9):e0255939. doi: 10.1371/journal.pone.0255939 (PMC8409666; doi:10.1371/journal.pone.0255939)
Supplement: S2 Table — (DOCX) [file pone.0255939.s002.docx]

**Supplementary Table 2: Performance of 3D UNet-based segmentation model on same test dataset of Dataset A**

| **Model** | **Lesion size (in voxels)** | **5-10** | **11-20** | **21-50** | **51-100** | **>100** | **Total** |
| --- | --- | --- | --- | --- | --- | --- | --- |
|  | **Total lesion count** | 21 | 41 | 49 | 36 | 32 | 179 |
| 3D-UNet | TP Count | 8 | 31 | 42 | 33 | 32 | 146 |
|  | Sensitivity | 0.381 | 0.756 | 0.857 | 0.917 | 1 | 0.816 |
|  | FP Count | 97 | 46 | 31 | 6 | 3 | 183 |
|  | FDR | 0.924 | 0.597 | 0.425 | 0.154 | 0.086 | 0.556 |
|  | Dice | 0.091 | 0.364 | 0.438 | 0.650 | 0.729 | 0.588 |
| 3D-UNet  + RF | TP Count | 7 | 21 | 35 | 29 | 31 | 123 |
|  | Sensitivity | 0.333 | 0.512 | 0.714 | 0.806 | 0.969 | 0.687 |
|  | FP Count | 28 | 23 | 19 | 6 | 2 | 78 |
|  | FDR | 0.800 | 0.523 | 0.352 | 0.171 | 0.061 | 0.388 |
|  | Dice | 0.178 | 0.415 | 0.446 | 0.624 | 0.731 | 0.625 |
