# Supplementary material for: Automatic segmentation of gadolinium-enhancing lesions in multiple sclerosis using deep learning from clinical MRI
Source: PLoS One. 2021 Sep 1;16(9):e0255939. doi: 10.1371/journal.pone.0255939 (PMC8409666; doi:10.1371/journal.pone.0255939)
Supplement: S3 Table — (DOCX) [file pone.0255939.s003.docx]

**Supplementary Table 3: p-values for gadolinium-enhancing lesion detection between 2D-UNet and 2D-Unet + RF using different loss functions**

| **Gadolinium-enhancing lesion detection matrices used to calculate p-values** | **Loss function used for the model training** | | | | |
| --- | --- | --- | --- | --- | --- |
|  | **Dice**  **coefficient**  **loss** | **Bootstrapping**  **cross entropy**  **(K= 256)** | **Bootstrapping**  **cross entropy**  **(K= 256 X 6)** | **Bootstrapping**  **cross entropy**  **(K= 256 X 12)** | **Cross entropy** |
| Sensitivity | 0.037 | 0.016 | 0.031 | 0.012 | 0.064 |
| False detection ratio (FDR) | 2.11 X 10^-5^ | 1.66 X 10^-5^ | 1.22 X 10^-5^ | 1.49 X 10^-14^ | 1.49 X 10^-7^ |
