# Supplementary material for: Automatic segmentation of gadolinium-enhancing lesions in multiple sclerosis using deep learning from clinical MRI
Source: PLoS One. 2021 Sep 1;16(9):e0255939. doi: 10.1371/journal.pone.0255939 (PMC8409666; doi:10.1371/journal.pone.0255939)
Supplement: S4 Table — (DOCX) [file pone.0255939.s004.docx]

**Supplementary Table 4: p-values for gadolinium-enhancing lesion detection for 2D-Unet + RF between cross entropy loss and other loss functions**

| **Gadolinium-enhancing lesion detection matrices used to calculate p-values** | **Dice**  **coefficient**  **loss** | **Bootstrapping**  **cross entropy**  **(K= 256)** | **Bootstrapping**  **cross entropy**  **(K= 256 X 6)** | **Bootstrapping**  **cross entropy**  **(K= 256 X 12)** |
| --- | --- | --- | --- | --- |
| Sensitivity | 0.753 | 0.456 | 0.8371 | 0.023 |
| False detection ratio (FDR) | 0.912 | 0.012 | 0.010 | 0.623 |
| Dice coefficient | 0.0258 | 0.5349 | 0.7211 | 5.97 X 10^-6^ |
