# Supplementary material for: Automatic segmentation of gadolinium-enhancing lesions in multiple sclerosis using deep learning from clinical MRI
Source: PLoS One. 2021 Sep 1;16(9):e0255939. doi: 10.1371/journal.pone.0255939 (PMC8409666; doi:10.1371/journal.pone.0255939)
Supplement: S5 Table — The diagonal elements represent the number of images for which the predicted lesion count is equal to the true lesion count. (DOCX) [file pone.0255939.s005.docx]

**Supplementary Table 5: Confusion matrix lesion count results on Dataset B for different magnetic fields. The diagonal elements represent the number of images for which the predicted lesion count is equal to the true lesion count**.

| Magnetic field strength (Total Number of Scans) | | 1 Tesla (30) | | | 1.5 Tesla (954) | | | 3 Tesla (1860) | | |
| --- | --- | --- | --- | --- | --- | --- | --- | --- | --- | --- |
| Overall Accuracy | | 86.67% | | | 82.2% | | | 90.7% | | |
|  | | **True lesion count** | | | **True lesion count** | | | **True lesion count** | | |
|  | | **0 lesion count** | **1 lesion count** | **≥2 lesion count** | **0 lesion count** | **1 lesion count** | **≥2 lesion count** | **0 lesion count** | **1 lesion count** | **≥2 lesion count** |
| Predicted lesion count | **0 lesion count** | 24 (85.7%) | 0  (0.0%) | 0 (0.0%) | 730 (83.3%) | 10  (23.8%) | 0  (0.0%) | 1626 (92.6%) | 28  (42.4%) | 7  (17.9%) |
|  | **1 lesion count** | 2 (7.1%) | 1 (100.0%) | 0 (0.0%) | 115 (13.1%) | 26 (61.9%) | 7  (19.4%) | 116  (6.6%) | 37  (56.1%) | 8  (20.5%) |
|  | **≥2 lesion count** | 2 (7.1%) | 0  (0.0%) | 1 (100.0%) | 31 (3.54%) | 6 (14.3%) | 29  (80.6%) | 13  (0.7%) | 1  (1.5%) | 24  (61.5%) |
