# Supplementary material for: Automatic segmentation of gadolinium-enhancing lesions in multiple sclerosis using deep learning from clinical MRI
Source: PLoS One. 2021 Sep 1;16(9):e0255939. doi: 10.1371/journal.pone.0255939 (PMC8409666; doi:10.1371/journal.pone.0255939)
Supplement: S6 Table — The diagonal elements represent the number of images for which the predicted lesion count is equal to the true lesion count. (DOCX) [file pone.0255939.s006.docx]

**Supplementary Table 6: Confusion matrix lesion count results on Dataset B for different vendors. The diagonal elements represent the number of images for which the predicted lesion count is equal to the true lesion count.**

| Vendors (number of scans) | | Siemens (2741) | | | Philips Medical Systems (103) | | |
| --- | --- | --- | --- | --- | --- | --- | --- |
| Overall Accuracy | | 88.3% | | | 76.6% | | |
|  |  | **True lesion count** | | | **True lesion count** | | |
|  |  | **0 lesion count** | **1 lesion count** | **≥2 lesion count** | **0 lesion count** | **1 lesion count** | **≥2 lesion count** |
| Predicted lesion count | **0 lesion count** | 2309  (89.8%) | 37  (35.6%) | 7  (10.4%) | 71  (79.8%) | 1  (20.0%) | 0  (0.0%) |
|  | **1 lesion count** | 219  (8.5%) | 60  (57.7%) | 10  (14.9%) | 14  (15.7%) | 4  (80.0%) | 5  (55.6%) |
|  | **≥2 lesion count** | 42  (1.6%) | 7  (6.7%) | 50  (74.6%) | 4  (4.5%) | 0  (0.0%) | 4  (44.4%) |
