# Supplementary material for: Automatic segmentation of gadolinium-enhancing lesions in multiple sclerosis using deep learning from clinical MRI
Source: PLoS One. 2021 Sep 1;16(9):e0255939. doi: 10.1371/journal.pone.0255939 (PMC8409666; doi:10.1371/journal.pone.0255939)
Supplement: S7 Table — The diagonal elements represent the number of images for which the predicted lesion count is equal to the true lesion count. (DOCX) [file pone.0255939.s007.docx]

**Supplementary Table 7: Confusion matrix lesion count results on Dataset B for different acquisition formats of T1 pre-contrast sequences. The diagonal elements represent the number of images for which the predicted lesion count is equal to the true lesion count.**

| Different acquisition format of T1 pre-contrast sequences | | 2D acquisition (1187) | | | 3D acquisition (1659) | | |
| --- | --- | --- | --- | --- | --- | --- | --- |
| Overall Accuracy | | 83.9% | | | 90.5% | | |
|  |  | **True lesion count** | | | **True lesion count** | | |
|  |  | **0 lesion count** | **1 lesion count** | **≥2 lesion count** | **0 lesion count** | **1 lesion count** | **≥2 lesion count** |
| Predicted lesion count | **0 lesion count** | 931  (84.9%) | 12  (24.5%) | 0  (0.0%) | 1450  (92.7%) | 26  (43.3%) | 7  (20.6%) |
|  | **1 lesion count** | 130  (11.9%) | 31  (63.3%) | 8  (19.0%) | 104  (6.6%) | 33  (55.0%) | 7  (20.6%) |
|  | **≥2 lesion count** | 35  (3.2%) | 6  (12.2%) | 34  (81.0%) | 11  (0.7%) | 1  (1.7%) | 20  (58.8%) |
