# Supplementary material for: Automatic segmentation of gadolinium-enhancing lesions in multiple sclerosis using deep learning from clinical MRI
Source: PLoS One. 2021 Sep 1;16(9):e0255939. doi: 10.1371/journal.pone.0255939 (PMC8409666; doi:10.1371/journal.pone.0255939)
Supplement: S9 Table — The diagonal elements represent the number of images for which the predicted lesion count is equal to the true lesion count. (DOCX) [file pone.0255939.s009.docx]

**Supplementary Table 9: Confusion matrix lesion count results on Dataset B for different sequences for pre and post-contrast T1-weighted scans. The diagonal elements represent the number of images for which the predicted lesion count is equal to the true lesion count**

| Sequences for pre and post-contrast T1-weighted scans | | Same Sequences (1018) | | | Different Sequences (1828) | | |
| --- | --- | --- | --- | --- | --- | --- | --- |
| Overall Accuracy | | 84.1% | | | 89.9 % | | |
|  |  | **True lesion count** | | | **True lesion count** | | |
|  |  | **0 lesion count** | **1 lesion count** | **≥2 lesion count** | **0 lesion count** | **1 lesion count** | **≥2 lesion count** |
| Predicted lesion count | **0 lesion count** | 801  (85.1%) | 10  (25.6%) | 0  (0.0%) | 1580  (91.9%) | 28  (40.0%) | 7  (18.4%) |
|  | **1 lesion count** | 110  (11.7%) | 25  (64.1%) | 8  (21.1%) | 124  (7.2%) | 39  (55.7%) | 7  (18.4%) |
|  | **≥2 lesion count** | 30  (3.2%) | 4  (10.3%) | 30  (78.9%) | 16  (0.9%) | 3  (4.3%) | 24  (63.2%) |
